# Le paludisme aujourd'hui

**DOI:** 10.48327/mtsi.v3i2.2023.375

**Published:** 2023-05-15

**Authors:** Martin DANIS

**Affiliations:** Professeur des universités-Praticien hospitalier émérite, CHU Pitié-Salpêtrière, Sorbonne Université, Faculté de médecine de Paris; Membre titulaire émérite de l'Académie de médecine; * Actes du Colloque - Centenaire de la mort d'Alphonse Laveran. 24 novembre 2022, Paris / Proceedings of the Conference - Centenary of the death of Alphonse Laveran. 24 November 2022, Paris

**Keywords:** Alphonse Laveran, Paludisme, Incidence, Mortalité, Monde, *Plasmodium falciparum*, Lutte antipaludique, Alphonse Laveran Malaria, Incidence, Mortality, World, P*lasmodium falciparum*, Malaria control

## Abstract

Le paludisme, maladie parasitaire dont l'agent pathogène a été découvert par Alphonse Laveran en 1880 en Algérie dans le sang de patients fébriles, reste en 2022 l'endémie des pays tropicaux et subtropicaux la plus fréquente. Dans son dernier « Rapport sur le paludisme dans le monde » disponible en novembre 2021, l'OMS rend compte très en détail des données recueillies sur le terrain en 2019-2020, de leur évolution depuis 20 ans et des mesures à prendre pour tenter de mieux contrôler cette endémie meurtrière. Le nombre de cas de paludisme est estimé à 232 millions en 2019 dans 87 pays d'endémie palustre, soit une baisse par rapport aux 245 millions de 2000. La région Afrique de l'OMS représente à elle seule 94 % des cas et les infections dues à l'espèce *Plasmodium falciparum* les plus fréquentes et les plus graves. En l'absence de prise en charge rapide chez les enfants de moins de 5 ans, le décès est à craindre. Au niveau mondial, le nombre de décès dus au paludisme a baissé de façon régulière sur la période 2000-2019, passant de 897 000 en 2000 à 568 000 en 2019. Près de 95 % des décès ont été enregistrés dans 31 pays, essentiellement d'Afrique subsaharienne. Dans les autres régions OMS, Asie du Sud-Est en particulier, le nombre de décès dus au paludisme a diminué de 74 %, avec 35 000 décès en 2000 contre 9000 en 2019. Le contrôle du paludisme dans le monde, son éradication peut-être, sont envisageables, à condition de dynamiser des campagnes d'information grand public et d'arracher des financements considérables.

## Abstract (en)

Malaria, a parasitic disease the pathogen of which was discovered by Alphonse Laveran in 1880 in the blood of febrile patients, remains in 2022 the most frequent endemic disease in tropical and subtropical countries. In its latest “World Malaria Report” available in November 2021, the WHO deals in great detail with the data collected in the field in 2019-2020, their progression over the last 20 years, and the measures to be taken to try to better control this life-threatening endemic. The number of malaria cases is estimated at 232 million in 2019 in 87 endemic countries, down from 245 million in 2000. The WHO African Region alone accounts for 94% of cases and the most frequent and severe infections due to *Plasmodium falciparum* species. If children under the age of 5 are not treated promptly, they can die. Globally, the number of malaria deaths declined steadily over the period 2000-2019, from 897,000 in 2000 to 568,000 in 2019, with nearly 95% of deaths occurring in 31 countries, primarily in sub-Saharan Africa. In other WHO regions, including Southeast Asia, malaria deaths decreased by 74%, with 35,000 deaths in 2000 compared to 9,000 in 2019. Malaria can be controlled worldwide, and possibly eradicated, if public information campaigns are strengthened and sufficient funds are made available.

Puis-je évoquer en préliminaire mon premier contact avec la médecine militaire ? C’était en 1968, j'avais décidé de faire un service national au titre de la coopération. J'ai donc été envoyé à Marseille pour suivre la formation de l’École du Pharo. Excellent enseignement, pragmatique, donnant les marches à suivre pour mettre en œuvre seul, les bons gestes urgents. J'ai été affecté au sud de la Mauritanie, à Aïoun El Atrouss où j'ai rejoint un médecin commandant, en poste depuis plusieurs années, ayant eu des conflits aigus avec certains responsables de la ville. Il a sans doute été content d'avoir un médecin adjoint. Le paludisme n'est pas un problème majeur à Aïoun, sauf après des pluies abondantes qui font couler de l'eau dans l'oued et remplissent toutes les mares, avec pullulation de moustiques vecteurs.

Un drame familial survenu en France fin août avec décès et blessures graves, m'a obligé à demander et obtenir mon rapatriement à Paris, avec l'obligation de poursuivre un an de service militaire, d'abord à Vincennes, puis à Libourne à l’École nationale des officiers de réserve du Service de santé des armées. Muni d'un uniforme avec képi et d'un grade d'aspirant, sans avoir été mobilisé sur le paludisme depuis l’École du Pharo, j'ai été affecté à l’État-major de la 2^e^ DB à Saint-Germain-en-Laye.

J'aborde maintenant le « paludisme aujourd'hui », en commençant par vous donner quelques sources à consulter. Je vous recommande un article intitulé « A brief history of malaria » rédigé essentiellement par François Nosten, probablement le Français qui a le plus publié sur le paludisme (707 publications dans PubMed entre 1987 et 2013), initié et conseillé par Nicolas White, un professeur anglais à Bangkok, paludologue expert internationalement reconnu. Nosten travaille depuis plusieurs dizaines d'années à la frontière Thaïlande-Myanmar et a organisé la prise en charge des populations Karen fuyant la dictature des généraux birmans en créant des villages autogérés avec un poste médical clinique et biologique où de nouvelles combinaisons thérapeutiques antipaludiques ont été validées pour parer aux chimiorésistances émergentes. Cette récente publication de synthèse complète, disponible en ligne, fait un point détaillé et complet sur le paludisme, avec 45 références bibliographiques [9].

Depuis 2008, l'Organisation mondiale de la santé (OMS) publie tous les ans un « Rapport sur le paludisme dans le monde » disponible en ligne en fin d'année, qui rassemble les données de l'année précédente envoyées par le Programme national de lutte contre le paludisme de tous les pays d'endémie [10, 11]. Ces données sont analysées par des experts qui calculent une valeur basse et une valeur haute, sorte d'intervalle de confiance (Fig. 1). Les nombres de cas et de morts sont comparés à ceux figurant dans le rapport de l'année précédente. Outre l’évolution annuelle, l'analyse porte sur les trois années précédentes au cours desquelles l'absence de cas autochtone est mentionnée en bleu sur la carte (Fig. 2). Il y a généralement peu de changements sur cette carte d'un rapport sur l'autre. Le rapport daté de 2022 est un fichier qui fait près de 300 pages, extrêmement complet et compliqué à analyser en détail, illustré par des dizaines de courbes, histogrammes et cartes [10]. En 2020, une légère augmentation porte à 245 millions le nombre de cas de paludisme autochtones (*versus* 232 millions en 2019). Probablement que le Covid-19 a eu une petite part dans cette augmentation du nombre de cas ainsi que pour le nombre de décès (568 000 en 2019 à 625 000 en 2020) [10], en raison de la désorganisation de la prise en charge du paludisme comme le montrent 5 452 articles publiés cette année-là. La carte issue du rapport 2022^1^1Le rapport 2022, dont sont extraites les Figures 1 et 2, est paru quelques jours après la conférence. montre que tout le nord de notre planète et l'Australie, en blanc sur la carte, sont exempts de paludisme autochtone (Fig. 2). Les zones où le paludisme se transmet régulièrement sont en rouge, soit 84 à 86 pays dans le monde. Ainsi, 247 millions de cas (avec un intervalle de confiance de 224 à 276 millions) ont été rapportés dans 84 pays, avec 619 000 (entre 577 000 et 754 000) décès en 2021 [11]. Plus de 94 % des cas sont déclarés par 31 pays – subsahariens pour la plupart – impliquant plus d'un milliard d'habitants.

**Figure 1:**
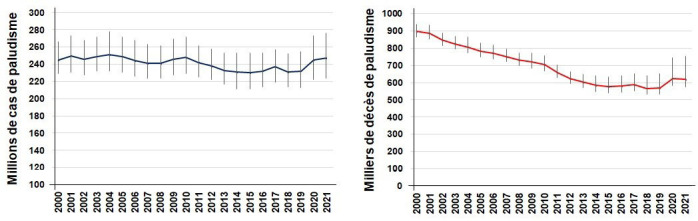
Incidence et mortalité par paludisme dans le monde depuis 2000 (d'après le Rapport de l'OMS sur le paludisme dans le monde [11]) Worldwide incidence and mortality of malaria since 2000 (based on WHO's World malaria report 2022 [11]

**Figure 2:**
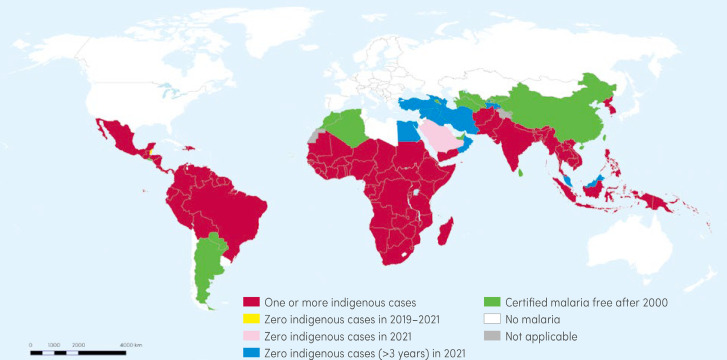
Paludisme dans le monde (d'après le Rapport de l'OMS sur le paludisme dans le monde [11]) Malaria throughout the world (based on WHO's World malaria report 2022 [11]

Le paludisme est une maladie infectieuse endémique dans la plupart des pays tropicaux – 86 pays concernés – depuis des centaines d'années. Elle frappe plus de 240 millions d'humains et en tue environ 600 000 par an, surtout des enfants de moins de 5 ans et essentiellement en Afrique subsaharienne. C'est donc un véritable drame, intolérable, une des maladies infectieuses les plus fréquentes dans le monde. Elle est prise en charge de façon intense avec une structure présente dans les pays endémiques visant à mobiliser et informer la population, à savoir l'initiative « Faire reculer le paludisme » (*Roll back malaria*). Ses objectifs sont de faire évoluer les outils (par exemple par la promotion des moustiquaires imprégnées d'insecticides distribuées gratuitement depuis plus de 10 ans) et de mieux financer la lutte [15], notamment grâce au Fonds mondial richement doté à la dernière refondation par ses organismes de tutelle.

La prévention et la lutte contre le paludisme s'appuient sur les centres de santé communautaire avec des actions comme celles menées par l'OPALS (Organisation panafricaine de lutte contre le SIDA) fondée par le Professeur Marc Gentilini. Auparavant dédiée au seul VIH, cette dernière est devenue en 2006 l'Organisation panafricaine de lutte pour la santé qui a adopté une nouvelle stratégie afin de protéger les jeunes générations grâce à des programmes au bénéfice de la mère et de l'enfant. Dans une localité proche de Kindia, en Guinée, j'avais été en contact avec un programme de réhabilitation de tous les centres de santé communautaire. Ils disposaient tous d'un laboratoire fonctionnel pourvu d’électricité, avec un responsable formé et ayant la confiance de la population du village. Ils étaient réapprovisionnés régulièrement en tests de diagnostic rapide sur bandelette (TDR), en combinaisons thérapeutiques à base d'arté-misinine (CTA^2^2En anglais ACT.) et en ressources pour orienter le diagnostic plutôt vers une infection bactérienne. Les consultations les plus nombreuses concernent des femmes, souvent enceintes (traitement préventif systématique du paludisme), accompagnées d'un enfant de 3-4 ans fébrile et malnutri, avec un TDR paludisme positif, qui en l'absence de signes de gravité, sera traité immédiatement en 3 jours par un CTA, en insistant sur l'importance de l'utilisation de la moustiquaire imprégnée d'insecticide. Si nécessaire, les patients sont évacués en urgence à l'hôpital de Kindia. Revu un an plus tard, le même responsable de ce centre de santé constatait une diminution considérable des cas de paludisme.

Le paludisme en France est surveillé par les laboratoires de référence du paludisme : Sandrine Houzé qui coordonne le Centre national de référence du paludisme à Bichat-Claude Bernard, Franck de Laval du Service de santé des armées qui travaille à Marseille sur l'examen très détaillé des résistances, et Lise Musset, à l'Institut Pasteur de Cayenne en Guyane où le paludisme se transmet encore. Lise Musset pilote un projet qui vise à éliminer le paludisme de Guyane en se concertant avec le Suriname, le Guyana et le Brésil. Le paludisme est présent en amont des fleuves Maroni et Oyapock où il est difficile à maîtriser, et en pleine forêt chez les orpailleurs clandestins. Il diminue cependant tous les ans (212 cas en 2019 contre 546 en 2018).

En France hexagonale (près de 5 000 cas chaque année), des précisions sont disponibles pour plus de 2300 cas de paludisme d'importation en 2021, dont 81 % de voyageurs, 10 % de résidents en zone d'endémie et 3 % de militaires [12]. Huit cas autochtones ont été déclarés, principalement dus au paludisme d'aéroport lié à la présence d'anophèles transportés par avion depuis les pays tropicaux. Si la cabine est désinsectisée par pulvérisation d'insecticides rémanents après sa fermeture, la cale ne l'est jamais. À l'ouverture de celle-ci, des anophèles éventuellement porteurs de *Plasmodium* s’échappent et peuvent piquer les personnes qui circulent dans l'aéroport. Exceptionnels, ces cas autochtones n'entraînent pas de transmission pérenne, même si des anophèles locaux sont impliqués [1, 14]. Cependant, les cas dits autochtones peuvent résulter d'une contamination hospitalière : dans le même service un patient traité pour un paludisme peut être à l'origine d'une transmission nosocomiale après des échanges mal maîtrisés de prélèvements [4].

En 2006-2007, *P. falciparum,* qui a montré depuis le début de l'utilisation des médicaments antipaludiques sa capacité à exprimer des souches mutantes résistantes à la chloroquine (années 1960-1970), à la sulfadoxine-pyrimé-thamine (années 1980), à la méfloquine (années 2000), disparaît plus lentement dans le sang sous artémisinine [6, 13]. Cette molécule complexe, sesquiterpène lactone peroxyde, a été isolée en 1973 en Chine des feuilles d'une armoise (*Artemisia annua,* Youyou Tu, prix Nobel 2015). Ce nouvel antipaludique agit habituellement rapidement et puissamment sur les plasmodies intra-érythrocytaires, mais sa demi-vie sanguine est courte. Ses formes orales, l'arté-sunate, l'artéméther, la dihydroartémisinine, sont associées à un autre antipaludique ayant un mode d'action différent et une longue demivie qui, en principe, doit retarder l'apparition de résistances. Les CTA sont indiquées partout dans le monde pour un accès non compliqué à *P. falciparum.* Une forme injectable par voie intraveineuse de l'artésunate a été fabriquée en Chine, pour le traitement urgent des accès graves à *P. falciparum.* Deux grandes études comparatives artésunate *versus* quinine dans des paludismes graves à *P. falciparum* essentiellement chez des adultes en Asie pour l'une [3], et chez des enfants en Afrique pour l'autre [2], ont montré significativement moins de morts dans le bras artésunate. L'OMS a préqualifié cet artésunate injectable aux bonnes pratiques de fabrication dans l'usine chinoise de Guilin. L'Agence française du médicament a validé une ATU nominative différée avec un stockage dans la « Pharmacie du Médicament » des hôpitaux qui le demande du Malacef^®^ (artésunate 60 mg), permettant une administration en urgence avec un relais par un CTA oral dès que le patient peut absorber des comprimés. Tous les accès graves à *P. falciparum* doivent être traités par l'artésunate parentéral en première intention [12]. Le taux de mortalité reste cependant à 5 %. Les marqueurs génétiques des mutants résistants à l'artémisinine dans le système K13 ont été identifiés et sont déjà présents dans quelques pays d'Afrique de l'Est [13], sans conséquence pour le moment sur l'efficacité des CTA grâce à la persistance d'activité des autres composants, contrairement à ce qui a été observé en Asie du Sud-Est dans le bassin du Mékong où tous les composants des CTA sont devenus inactifs [5]. Si une évolution semblable se dessine en Afrique, elle constituera un véritable drame pour la lutte antipaludique.

Pour les vaccinations, désormais conseillées par l'OMS, je vous renvoie aux travaux de Marie Mura et à sa communication [7, 8].

Dans sa précédente Journée mondiale du paludisme en avril 2021, l'OMS avait pour objectif d’éliminer la maladie dans 25 pays d'endémie. Un an plus tard, lors de celle de 2022, elle ne cible plus que l'amélioration des moyens de lutte. Cette maladie infectieuse transmise par des piqûres de moustiques reste donc un drame absolu, et l'infection endémique la plus fréquente dans le monde est insupportable. Le paludisme doit être pris en charge, mais il faut vraiment une implication de toutes les parties prenantes et que les financements nécessaires soient disponibles (il manquait 3 milliards de dollars pour financer la lutte en 2022).

## Remerciements

Merci aux Dr Jean-Philippe Chippaux et Jean-Paul Boutin pour leur contribution précieuse à la mise en forme de cette communication.

## Liens D'intérêts

L'auteur ne déclare aucun lien d'intérêt.

## References

[1] Armengaud A, Legros F, D'Ortenzio E, Quatresous I, Barre H, Houze S, Valayer P, Fanton Y, Schaffner F (2008). A case of autochthonous Plasmodium vivax malaria, Corsica, August 2006. Travel Med Infect Dis.

[2] Dondorp AM, Fanello CI, Hendriksen IC, Gomes E, Seni A, Chhaganlal KD, Bojang K, Olaosebikan R, Anunobi N, Maitland K, Kivaya E, Agbenyega T, Nguah SB, Evans J, Gesase S, Kahabuka C, Mtove G, Nadjm B, Deen J, Mwanga-Amumpaire J, Nansumba M, Karema C, Umulisa N, Uwimana A, Mokuolu OA, Adedoyin OT, Johnson WB, Tshefu AK, Onyamboko MA, Sakulthaew T, Ngum WP, Silamut K, Stepniewska K, Woodrow CJ, Bethell D, Wills B, Oneko M, Peto TE, von Seidlein L, Day NP, White NJ, AQUAMAT group (2010). Artesunate versus quinine in the treatment of severe falciparum malaria in African children (AQUAMAT): an open-label, randomized trial. Lancet.

[3] Dondorp AM, Nosten F, Stepniewska K, Day N, White N, South East Asian Quinine Artesunate Malaria Trial (SEAQUAMAT) group (2005). Artesunate versus quinine for treatment of severe falciparum malaria: a randomised trial. Lancet.

[4] Gruell H, Hamacher L, Jennissen V, Tuchscherer A, Ostendorf N, Löffler T, Hallek M, Kochanek M, Tannich E, Böll B, Fätkenheuer G (2017). On Taking a Different Route: An Unlikely Case of Malaria by Nosocomial Transmission. Clin Infect Dis.

[5] Hanboonkunupakarn B, Tarning J, Pukrittayakamee S, Chotivanich K (2022). Artemisinin resistance and malaria elimination: Where are we now?. Front Pharmacol.

[6] Hanboonkunupakarn B, White NJ (2022). Advances and roadblocks in the treatment of malaria. Br J Clin Pharmacol.

[7] Mura M (2023). Vaccination contre le paludisme. Med Trop Sante Int.

[8] Mura M, Lu P, Atre T, Bolton JS, Duncan EH, Chaudhury S, Bergmann-Leitner ES (2022). Immunoprofiling Identifies Functional B and T Cell Subsets Induced by an Attenuated Whole Parasite Malaria Vaccine as Correlates of Sterile Immunity. Vaccines (Basel).

[9] Nosten F, Richard-Lenoble D, Danis M (2022). A brief history of malaria. Presse Med.

[10] OMS (2021). World malaria report 2021. Organisation mondiale de la santé, Genève.

[11] OMS (2022). World malaria report 2022. Organisation mondiale de la santé, Genève.

[12] Roussel C, Ndour PA, Kendjo E, Larréché S, Taieb A, Henry B, Lebrun-Vignes B, Chambrion C, Argy N, Houzé S, Mouri O, Courtin D, Angoulvant A, Delacour H, Gay F, Siriez JY, Danis M, Bruneel F, Bouchaud O, Caumes E, Piarroux R, Thellier M, Jauréguiberry S, Buffet P, French Artesunate Working Group (2021). Intravenous Artesunate for the Treatment of Severe Imported Malaria: Implementation, Efficacy, and Safety in 1391 Patients. Clin Infect Dis.

[13] Stokes BH, Dhingra SK, Rubiano K, Mok S, Straimer J, Gnädig NF, Deni I, Schindler KA, Bath JR, Ward KE, Striepen J, Yeo T, Ross LS, Legrand E, Ariey F, Cunningham CH, Souleymane IM, Gansané A, Nzoumbou-Boko R, Ndayikunda C, Kabanywanyi AM, Uwimana A, Smith SJ, Kolley O, Ndounga M, Warsame M, Leang R, Nosten F, Anderson TJ, Rosenthal PJ, Ménard D, Fidock DA (2021). Plasmodium falciparum K13 mutations in Africa and Asia impact artemisinin resistance and parasite fitness. Elife.

[14] Tseroni M, Georgitsou M, Baka A, Pinaka O, Pervanidou D, Tsironi M, Bleta P, Charvalakou M, Psinaki I, Dionysopoulou M, Legaki A, Vakali A, Patsoula E, Vassalou E, Bellou S, Diamantopoulos V, Georgakopoulou T, Mouchtouri V, Tsiodras S, Middleton N, Charalambous A, Raftopoulos V, Hadjichristodoulou C (2020). The Importance of an Active Case Detection (ACD) Programme for Malaria among Migrants from Malaria Endemic Countries: The Greek Experience in a Receptive and Vulnerable Area. Int J Environ Res Public Health.

[15] White NJ, Day NPJ, Ashley EA, Smithuis FM, Nosten FH (2022). Have we really failed to roll back malaria?. Lancet.

